# Mental health consequences during alerting situations and recovering to a new normal of coronavirus epidemic in 2019: a cross-sectional study based on the affected population

**DOI:** 10.1186/s12889-021-11550-w

**Published:** 2021-08-03

**Authors:** Qian Zhang, Rujun Zheng, Yan Fu, Qianqian Mu, Junying Li

**Affiliations:** 1 West China School of Medicine/West China School of Nursing, Sichuan University, Chengdu, 610041 China; 2 Department of Thoracic Oncology, West China Hospital, Sichuan University, No. 37, Chengdu, 610041 China

**Keywords:** COVID-19, PTSD, Death anxiety, Psychological, Mental

## Abstract

**Background:**

As a major virus outbreak in the twenty-first century, the coronavirus disease 2019 (COVID-19) pandemic has caused unprecedented hazards to mental health globally.

**Methods:**

We performed a cross-sectional study based on the results of an online survey. The survey was conducted 1 month after the outbreak (February 18–29, 2020) and repeated at the time of resuming activity (April 8–14, 2020). The 15-item Death Anxiety Scale (T-DAS) was used to assess the degree of death anxiety, and the Chinese version of PTSD checklist-civilian version (PCL-C), for PTSD symptom clusters. Through convenient sampling, a total of 7678 cases were collected.

**Results:**

Our findings showed that even after the lockdown was lifted, the prevalence of the symptoms of post-traumatic stress disorder (PTSD) and death anxiety remained significantly high in the general population affected by the outbreak. Regression model analysis showed that PTSD was significantly associated with age > 50 years, contact history/living community, poor health status of participants, past traumatic experience (PTE), and medical occupation. Moreover, death anxiety mediated the relationship between life-threatening PTE and PTSD, indicating that reducing death anxiety could buffer the negative effects of PTE on PTSD.

**Conclusions:**

Despite the lifting of the lockdown, long-term adverse psychological effects remain in the affected general population. The management of mental health after major public health events is important, and high-risk groups such as the elderly and healthcare workers should receive targeted interventions. In addition, the study suggests that methods for alleviating death anxiety must be included in plans to manage the psychological impact of public health emergencies.

## Background

The COVID-19 pandemic has had an unprecedented impact on all societies worldwide, and the World Health Organization (WHO) declared the COVID-19 outbreak an international public health emergency on January 30, 2020 [1]. The rapid spread of large-scale infectious diseases in a short period often generates fear among individuals and affects their mental health [2–7]. Unlike the situation during the severe acute respiratory syndrome (SARS) outbreak in 2003, when communication technology was still in its infancy, the reach of communication technology at present is far greater. Data on daily number of cases and deaths, and real-time information from the frontlines regarding epidemic prevention contributed to the intensification and magnification of negative emotions regarding the novel coronavirus, such as fear and death anxiety, among people [2, 8, 9]. Continued exposure to an epidemic setting can induce an acute stress response or even post-traumatic stress response (PTSD).

Numerous studies have been conducted on the prevalence of PTSD after pandemics. However, the evolution of different stages (the initial stage, the lockdown stage, the mitigation stage) of PTSD remains controversial. During the initial stage, the 2019 coronavirus influenza pandemic caused prolonged exposure to stress, and the incidence of PTSD was associated with stress levels in different populations. Liang et al. assessed the mental health of adolescents in the early stages of the outbreak and found that 14.4% of young people had PTSD-related symptoms [10]. A study in China found that 9.8% of health workers had PTSD symptoms [11]. In the US, a survey of young people of age 18–30 years showed that 31.8% had symptoms of PTSD [12]. During the lockdown stage, governments required citizens to confine themselves to their homes and prohibited unnecessary travel in an effort to contain the outbreak. Passavanti et al., through a multicenter study, pointed out that more than half of the researchers reported PTSD [13]. An Indian study found that during the blockade, the incidence of mental illness increased by eight to ten times [14]. During the mitigation phase, as the severity of the outbreak lessened, several countries began to lift the mandatory lockdowns. In this regard, Eldhuis et al. found through follow-up observation that PTSD symptoms decreased with the remission of the epidemic19. However, Gonzalez-Sanguino et al. looked at 3480 ordinary Spaniards and found that the psychological effects of the epidemic, such as anxiety, were not relieved during the stable phase [15].

In addition, the 2019 coronavirus influenza pandemic led to the fear of death among people due to prolonged exposure to stress [2, 8, 9, 16, 17]. Death anxiety refers to the personal concern regarding existence and death in the face of threatening events, and it is a type of negative emotion [16, 18]. Curșeu et al. pointed out that death anxiety could be further aggravated in the presence of anxiety and negativity among the public in the face of an epidemic [16]. A survey conducted in Poland by Chodkiewicz et al. revealed that as a result of the epidemic, a large proportion of their respondents had symptoms such as anxiety and depression (40%) and suicidal thoughts (24%) [17]. Continued exposure to such conditions can induce an acute stress response or even post-traumatic stress response (PTSD) [16, 17].

It should be noted that most of these investigations have been conducted at a single point in time, with few studies investigating the evolution of the psychological and spiritual effects in the general population after deregulation in countries first affected by the COVID-19 pandemic. The current investigation is a cross-sectional study assessing the impact of the pandemic on the mental health of the general population at two points in time: 1 month after the outbreak and after the lifting of the lockdown (3 months after the outbreak). We analyzed the potential predictors of PTSD and the psychological effects of the epidemic the general population at different times. In addition, we developed a new mediation model to account for individual differences in sensitivity to the transition from past traumatic experience (PTE) to PTSD.

## Methods

Ethical approval for the study protocol was provided by the Biomedical Ethics Review Committee of West China Hospital of Sichuan University, protocol number 2020(930). All participants provided informed consent prior to participation in this study.

### Participants

This investigation was designed as a cross-sectional study of the results obtained from an online survey, which was first conducted 1 month after the outbreak (February 18–29, 2020) and repeated again when the lockdown was lifted (April 8–14, 20,202). Because it was not feasible to do a community-based national sampling survey during the lockdown period, we sought to collect the data online. The authors invited 89 volunteers from their own social networks to participate in the data collection, and all volunteers, in turn, posted/reposted one-page job posters in their own WeChat (WhatsApp) and Weibo (Twitter) Moments and Groups. This poster contained a brief introduction on the background, objective, procedures, voluntary nature of participation, declarations of anonymity and confidentiality, and notes for filling in the questionnaire, as well as the link and quick response (QR) code of the online questionnaire. Participants enrolled in the study completed the questionnaires by clicking on a link or scanning a QR code. Informed consent was given on the first page of the questionnaire, and participants had the right to refuse to participate in the research or to use their personal data for scientific research before the questionnaire began. The exclusion criteria were inability to read and write and refusal to participate in the study. Participants whose questionnaires were incomplete were also excluded from the study.

### Measures

#### Sociodemographic variables

An ad hoc questionnaire was created to collect data on standard demographic parameters, including gender, age, education level, marital status, health status, religious beliefs, and history of community contact with COVID-19 patients. Since past traumatic experience (PTE) is a strong predictor of PTSD, we also assessed for previous life-threatening trauma-related experiences (life-threatening experiences or bereavement experiences).

#### Post-traumatic stress disorder symptoms

We used the Chinese version of PTSD checklist-civilian version (PCL-C), which consists of 17 items [19]. PTSD was assessed on the basis of 4 symptom groups: (1) maintaining an avoidance attitude towards people; (2) re-experiencing various forms of symptom clusters, things, places, and situations related to traumatic events; (3) a state of cognitive decline and negative emotions; and (4) insomnia (difficulty falling asleep or awakening), irritability, shock, and other symptoms of increased alertness. Respondents were required to rate the severity of these PTSD symptoms over the past month, on a 5-point Likert scale ranging from 1 (not at all) to 5 (extremely). Total severity scores are the sum of all items, with a cut-point score of 38 used to identify patients exhibiting significant PTSD symptoms and dichotomized as “yes/no,” as in previous research on PCL-C [20, 21]. Previous studies have shown the reliable accuracy and validity of PCL-C in screening for PTSD, and the good internal consistency and effectiveness of the Chinese version of PCL-C have also been verified [20–22]. In this study, we screened patients according to whether or not they had the main symptom groups of PTSD and did not conduct a comprehensive clinical evaluation; therefore, the diagnosis of PTSD cannot be conclusively established in our study participants. The Cronbach α coefficient for the scale in this study was 0.969.

#### Death anxiety symptoms

The Templer–Death Anxiety Scale (DAS) was used to investigate the degree of death anxiety in the participants. This scale, which was originally developed by Templer, has been in use for the measurement of death anxiety in individuals and has been shown to have good reliability and validity [23, 24]. The Chinese version of DAS has been shown to have excellent internal consistency and convergent validity with the measurement of death anxiety [25]. In this scale, a total of 15 points are assigned to various domains: (1) emotion, which mainly pertains to subjective experiences and feelings in the emotional process and emotions related to death; (2) stress and pain, which represent the stress and pain of death caused by disease; (3) and time awareness (awareness regarding the transient and finite nature of life); and (4) cognition (emotional memory of death). A total score of 7 and above was indicative of high death anxiety, with higher scores indicating more severe death anxiety. In this study, Cronbach α coefficient for this scale was 0.724.

### Statistical analysis

The results of the online surveys were collected, and SPSS software version 25.0 was used for statistical analysis. Parameters for which the data conformed to normal distribution were expressed as mean ± standard deviation (x ± s), while countable data were expressed as frequency. The chi-square test was used to evaluate the difference between the first and second surveys in terms of the incidence of death anxiety and PTSD. Univariate analysis and multivariate binary logistic regression were performed to identify the risk factors of PTSD. First, a univariate analysis was performed to identify independent variables, followed by multivariate binary logistic regression. Among the variables identified in univariate analysis, those with a significance level of *P* < 0.10 were included in the multivariate analysis. Secondly, multivariate binary logistic regression was performed after the exclusion of confounding factors of each variable; the odds ratio (OR) was then corrected, and the statistical significance level of the final analysis was set at *P* < 0.05.

**Fig. 1:**
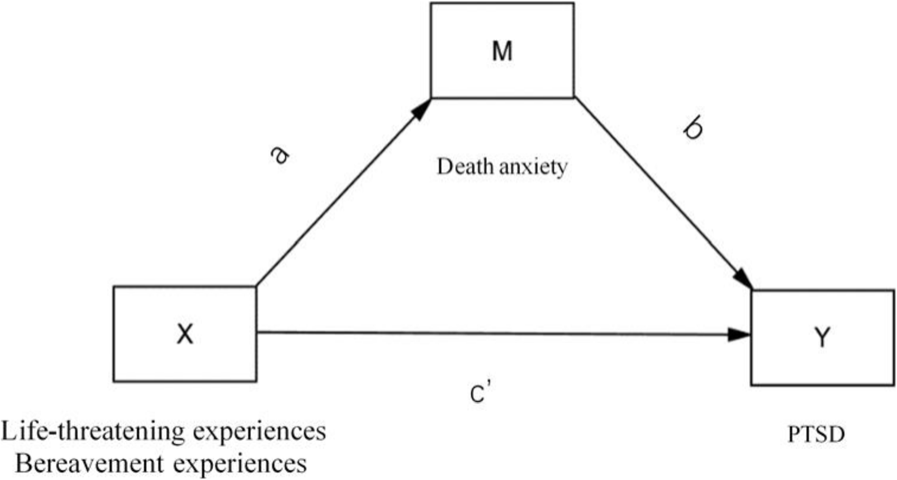
Simple and Mediated Relationship Model: Path diagram of the effects of past experiences on PTSD via death anxiety, after controlling for gender, age and occupation. X = independent variable, Past experience. Y = dependent variable, PTSD. M = mediator, Death anxiety. Ab = indirect effect. c’ = direct effect

Based on the theoretical model of stress, stressors (X), psychological mediators (M), and stress responses (Y) together constitute a complete process of spontaneous excitation [26]. A mediation analysis is a statistical approach that seeks to identify and explain factors that underlie an observed relationship between an independent variable (X, past experience) and a dependent variable (Y, PTSD), by including a third variable known as a mediator (M, death anxiety) (Fig. 1). We adopted the bootstrapping method employed by the PROCESS macro for SPSS (version 25). This method uses repeated sampling to obtain a bootstrap sample similar to the original sample, with a smaller likelihood of type I errors and freedom from assumptions of normality in the sample distribution. The criterion to determine the effect of mediation was the lack of a zero in the confidence interval of the indirect effect.

## Results

### Descriptive results

A total of 7771 participants completed the questionnaire. Among them, 84 participants refused to participate in the study, and 9 participants were excluded because their questionnaires were inconsistent. Thus, the final effective participation rate was 98.80% (7678). Among the individuals who completed the questionnaires, 6792 were women (88.46%) and 886 were men (11.54), with an average age of was 34.88 years (SD = 10.468). Among them, 5769 (75.14%) of the participants were medical staff, whereas the remaining 1974 (25.71%) were non-medical staff such as students, educators, and civil servants (Table 1).

**Table 1:** Respondents’ demographic characteristics

Variable	Outbreak phase N(%)	Work resumption phase N(%)	Total N(%)
Total	3762	3916	7678
Sex			
Female	3261 (86.7)	3531 (90.2)	6792 (88.46)
Male	501 (13.3)	385 (9.8)	886 (11.54)
Age			
< 25 y	512 (13.6)	919 (23.5)	1431 (18.64)
25 ~ 50 y	2553 (67.9)	2751 (70.3)	5304 (69.08)
> 50 y	697 (18.5)	246 (6.3)	943 (12.28)
Age Mean (SD)	37.91 (11.113)	31.98 (8.886)	34.88 (10.468)
Education			
Junior college or below	1634 (43.4)	1216 (31.1)	2850 (37.12)
Undergraduate	1899 (50.5)	2493 (63.7)	4392 (57.2)
Postgraduate and above	229 (6.1)	207 (5.3)	436 (5.68)
Occupation			
Non-medical staff	1432 (38.1)	477 (12.2)	1909 (24.86)
Medical staff	2330 (61.9)	3439 (87.8)	5769 (75.14)
Marital status			
Unmarried	707 (18.8)	1267 (32.4)	1974 (25.71)
Married	2875 (76.4)	2524 (64.5)	5399 (70.32)
Divorce	146 (3.9)	114 (2.9)	260 (3.39)
Widowed	34 (0.9)	11 (0.3)	45 (0.59)
Residence			
villages and towns	242 (6.4)	158 (4)	400 (5.21)
County-leve city	1040 (27.6)	713 (18.2)	1753 (22.83)
Prefecture-level city	855 (22.7)	2062 (52.7)	2917 (37.99)
Provincial capital city	1625 (43.2)	983 (25.1)	2608 (33.97)
Living community contact			
Yes	566 (15)	3269 (83.5)	3835 (49.95)
No	3196 (85)	647 (16.5)	3843 (50.05)
Religious belief			
Yes	332 (8.8)	3649 (93.2)	3981 (51.85)
No	3430 (91.2)	267 (6.8)	3697 (48.15)
Physical condition			
Good	3489 (92.7)	3780 (96.5)	7269 (94.67)
Worse	273 (7.3)	136 (3.5)	409 (5.33)
Life threatening event			
Yes	1001 (26.6)	3125 (79.8)	4126 (53.74)
No	2761 (73.4)	791 (20.2)	3552 (46.26)
Bereavement			
Yes	1716 (45.6)	2317 (59.2)	4033 (52.53)
No	2046 (54.4)	1599 (40.8)	3645 (47.47)

### Incidence of death anxiety and PTSD at the two time points

Between the first and second surveys, the percentage of people with high death anxiety in the two surveys rose from 48.1 to 53.2% (*P* < 0.001), whereas the incidence of PTSD increased from 6.43 to 8.60% (*P* < 0.001). Bivariate analysis revealed that both non-medical staff and medical staff had similarly high levels of death anxiety at the time of the outbreak (*P* = 0.851). At the second survey, once work was resumed, a significant difference was noted between the medical staff and non-medical staff in terms of the incidence of high death anxiety (*P* < 0.001; Tables 2 and 3).

**Table 2:** Difference between high mortality anxiety and PTSD in two time periods

Variable	Pearson Chi-Square	Outbreak phase (%)	Work resumption phase (%)	*P*
High death anxiety incidence	20.22	48.10	53.20	< 0.001
PTSD incidence	12.711	6.43	8.60	< 0.001

*Abbreviation*: *PTSD* Post-traumatic stress disorder

**Table 3:** Differences in death anxiety of different occupations in two periods

Variable	Non-medical staff	Medical staff	Pearson Chi-Square	*P*
Outbreak phase had (%)	48.3	47.9	0.035	0.851
Work-resumption phahadHDA (%)	45.3	54.3	13.646	< 0.001

*Abbreviation*: *HDA* High death anxiety

### Influencing factors of PTSD

We performed regression analysis while accounting for confounding factors in order to identify the predictors of PTSD (Table 4). Multivariate analysis revealed the following as risk factors associated with PTSD: age > 50 y (vs. age < 50 y; OR, 0.535; 95% CI, 0.366 ~ 0.783; *P* = 0. 001), medical staff (vs. non-medical staff; OR, 1.301; 95% CI 1.041 ~ 1.626; *P* = 0.021), history of community contact (vs. no contact history; OR, 1.353; 95% CI, 1.093 ~ 1.674; *P* = 0.006), poor health status of the participants (vs. good health; OR, 1.923; 95% CI, 1.407 ~ 2.628; *P* = 0.001), and the history of past life-threatening events (vs. no such history; OR, 1.570; 95% CI, 1.301 ~ 1.895; *P* < 0.001).

**Table 4:** Multivariate binary logistic regression analysis to determine risk factors of PTSD

Variable	OR (95% CI)	*P* value
Sex		
Female	1[Reference]	NA
Male	1.206 (0.894 ~ 1.627)	0.219
Age		
< 50 y	1[Reference]	NA
> 50 y	**0.535 (0.366 ~ 0.783)**	**0.001**
Education		
Junior college or below	1[Reference]	0.487
Undergraduate	1.044 (0.857 ~ 1.271)	0.667
Postgraduate and above	1.266 (0.861 ~ 1.861)	0.231
Occupation		
Non-medical staff	1[Reference]	NA
Medical staff	**1.301 (1.041 ~ 1.626)**	**0.021**
Marital status		
Unmarried	1[Reference]	0.338
Married	0.825 (0.641 ~ 1.062)	0.135
Divorce	0.854 (0.509 ~ 1.434)	0.551
Widowed	1.501 (0.513 ~ 4.391)	0.458
Residence		
Villages and towns	1[Reference]	0.327
County-level city	0.950 (0.614 ~ 1.471)	0.818
Prefecture-level city	1.054 (0.689 ~ 1.613)	0.809
Provincial capital city	0.870 (0.568 ~ 1.333)	0.522
Living community contact		
Yes	**1.353 (1.093 ~ 1.674)**	**0.006**
No	1[Reference]	NA
Physical condition		
Good	1[Reference]	NA
Worse	**1.923 (1.407 ~ 2.628)**	**< 0.001**
Past traumatic experience		
Yes	**1.570 (1.301 ~ 1.895)**	**< 0.001**
No	1[Reference]	NA
Bereavement		
Yes	1.061 (0.885 ~ 1.272)	0.523
No	1[Reference]	NA

*Abbreviation*: *PTSD* Post-traumatic stress disorder

### Mediating effect of death anxiety

The mediating role of death anxiety between PTE and PTSD was analyzed after controlling for gender, age, and occupation of participants using Model 4 of the plugin macro in SPSS. Table 5 shows the mediation analysis results which demonstrate that PTE directly and positively predicted PTSD (β = 0.3178, t = 3.1785, *p* < 0.001). Death anxiety had a significant predictive effect on PTSD (β = 0.2636, t = 19.3921, p < 0.001). Goodness of fit and significance of outcomes and predictors in the tests for the mediating effects of death anxiety are shown in Table 5. The significance of the mediating effect was tested with a bootstrap method in the sampling process. Table 6 shows the results of the bootstrap analysis. The upper and lower limits of the 95% confidence interval do not contain 0, which indicates that the total effects, direct effects (0.1218, 0.5138), and the indirect effects (0.1305,0.2371) of the mediation paths are all significant.

**Table 5:** Mediating effect of death anxiety

Regression equation	Predictor	Goodness of fit		Significance		
Outcome		R2	F	β	se	t
Death anxiety		0.1579	49.043			
	Previous trauma-related experiences			0.6936	0.95	7.3***
	Gender			1.2084	0.1277	9.4646***
	Age			−0.036	0.0041	−7.676***
	Occupation			−0.2483	0.1014	−2.45***
Post-traumatic stress disorder						
	Death anxiety			0.2636	0.0136	19.3921***
	Previous trauma-related experiences			0.3178	0.1	3.1785***
	Gender			−0.1248	0.1591	−0.7846***
	Age			−0.0177	0.0052	−3.3992***
	Occupation			0.2456	0.1211	2.0282***

*N* = 7678. All 95% confidence intervals of predictors were gained by conducting Bootstrap method, number of bootstrap samples are 5000. ***P<0.01

**Table 6:** Bootstrap analysis of multiple mediation effects

	Effect size	SE	95% CI	
			Lower limit	Upper limit
Direct effects	0.3178	0.1	0.1218	0.5138
Indirect effects	0.1828	0.0272	0.1305	0.2371

## Discussion

This study aims to elucidate the evolution of the impact on the psychological and mental health of the general population at various stages in a country that was first affected by the COVID-19 outbreak. As the study was conducted 1 month (outbreak phase) and 3 months (work resumption phase) after the epidemic outbreak, the full impact of COVID-19 on the mental health at different times was captured. This implies that our sample population could serve as a strong representative of epidemic-affected populations.

The prevalence of PTSD and death anxiety in the general population in the first month of COVID-19 outbreak was 6.43 and 48.10%, respectively, which increased significantly to 8.60 and 53.20% at 3 months after the outbreak. Previous studies among the general population in China during the COVID-19 outbreak have shown trends in psychological impact at different stages that were not significant [27, 28]. This difference between our study and previous studies may be attributed to the differences in the measurement tools. The Impact of Event Scale-Revised (IES-R) was used by previous studies, which is different from the one used in this study [27, 28]. Another possible explanation is that prolonged lockdown has serious adverse effects on mental health [29]. In addition, PTSD is distinct from anxiety and depression, and these differences might reflect the variations between the acute and long-term psychological effects triggered by an outbreak, which may be more pronounced among healthcare workers [30, 31].

Medical workers are at high risk of exposure to COVID-19 as compared to non-medical workers and are faced with high levels of stress [31, 32]. Studies have shown that in major public health emergencies, health workers are the main responders to outbreaks and that they are often left with more lasting and more complex psychological damage [33, 34]. In our study, we found that compared to the outbreak stage, the stable stage of the epidemic had a significantly higher psychological impact in medical workers than in non-medical workers. This may be the response of healthcare workers to actual or possible threats after close clinical contact with infected patients during their involvement in disease control [4, 6]. In addition, stress associated with close clinical contact with infected patients increases the likelihood of the formation of trauma-related memories [11, 35]. The finding highlights some implications for public health. First, as the psychological impact of the 2019 coronavirus epidemic continues, it is necessary to prioritize offering the best possible support for health workers in the current crisis. Secondly, the existence of traumatic medical memory has a long-term impact on medical workers, and it would be worthwhile to identify measures to reduce the traumatic medical memory in rescue.

Our study also revealed some of the factors that make individuals most vulnerable to the psychological consequences of the COVID-19 outbreak. Consistent with recent studies exploring the effects of the COVID-19 pandemic, our study showed that older age was associated with more symptoms of post-traumatic stress [15, 36]. This increased vulnerability may have a negative effect of a higher risk of infection in older people, making them more vulnerable than younger people [37, 38]. In addition, and in line with the findings of previous studies, we found that traumatic stress responses were greater in individuals with poor self-reported physical health [15, 39]. It is possible that the pre-existing burden of disease may fuel fears of widespread susceptibility [36, 40]. Individuals with trauma have been reported to exhibit a higher prevalence of PTSD as compared to those who have not experienced highly traumatic events (Wald chi-square = 10.96, df = 1, *P* = 0.0009) [41, 42]. The risk of PTSD is influenced by the number and type(s) of traumatic events experienced (e.g., violence, loss of loved ones, and unrequited affection) [42]. In this study, we focused on two past experiences, namely the history of bereavement and the history of life-threatening events. Among them, the participant’s own PTE were found to be significantly associated with the development of PTSD. People who had been in contact with the COVID-19 outbreak also had a high risk of PTSD. This finding might suggest that even the acquaintance or contact with a COVID-19 patient may be viewed by individuals as personalized trauma [43].

Besides exploring socio-demographic and work-related factors associated with PTSD development among general population, the novel contribution of the present study the establishment of a prediction model between PTE and PTSD. Mediation analysis confirmed that death anxiety was a predictor of progression to PTSD from a PTE. Although characteristics of PTE have been shown to influence the risk of PTSD, studies indicate that 89.7% of Americans are exposed to a significant traumatic event over the course of their lifetime, and only a small percentage develop mental illness [11, 44, 45]. Several other factors are involved in an individual’s susceptibility towards PTSD following PTE, which include gender, neuroticism, and family history of mental illness; these data are consistent with findings from Comings, Fullerton, and Stein [44, 46, 47]. Therefore, it is important to identify individual differences that may contribute to the development of the more severe manifestations of psychopathology following trauma [44]. While mature and systematic mode of death education may be provided in foreign countries, Chinese people are restricted by the life and death culture of “avoiding death while living,” and the theory and practice of death education are still in exploratory stages [48, 49]. The health system may not provide psychological support in terms of life education after an individual experiences a life-threatening trauma, and fear of death may trigger a traumatic emotional reaction when the individual is faced with a life-threatening event again [50–54]. In this study, the levels of death anxiety were found to be consistently high at both time points, with healthcare workers experiencing significantly higher levels of death anxiety 3 months after the outbreak than non-medical workers. This highlights the need to change the perception of death among the general population and provide them with effective life education. The more important message is that emergency plans for the improvement of psychological well-being, including life education, should be established for medical workers with traumatic experiences.

Our study has several limitations. First, the population surveyed in this study was not uniform and the baseline level of mental health of the participants before the survey was not accounted for. Second, when the content of the study was explained, some candidates refused participation because of the Chinese culture of avoiding the topic of death. It is unclear whether this rejection is in itself an indicator of higher levels of death anxiety. Finally, the participation rate of non-medical personnel was significantly lower than that of medical personnel, and again, we are unsure whether this lack of participation represents non-medical workers’ resistance to addressing the problem.

## Conclusion

This study shows the long-term psychological impact of the COVID-19 pandemic on the general population in China. Despite the initial crisis being overcome and the relaxation of containment measures, the negative effects of the outbreak, such as death anxiety and PTSD, continue to rise. These results underscore the importance of continuous attention to mental health and reveal key variables such as history of life-threatening events and occupation. In addition, it is more important to pay attention to the moderating effect of death anxiety on PTE and PTSD. These new findings may be taken into consideration when formulating a plan for the management of the psychological effects of public health emergencies and thereby provide effective interventions for key populations.

## Data Availability

The datasets used and/or analyzed during the current study are available from the corresponding author on reasonable request.

## Abbreviations

PTSD: Post-traumatic stress disorder
PTE: Past traumatic experience
COVID-19: Corona virus disease 2019
